# Recommendations for environmental risk assessment of gene drive applications for malaria vector control

**DOI:** 10.1186/s12936-022-04183-w

**Published:** 2022-05-25

**Authors:** John B. Connolly, John D. Mumford, Debora C. M. Glandorf, Sarah Hartley, Owen T. Lewis, Sam Weiss Evans, Geoff Turner, Camilla Beech, Naima Sykes, Mamadou B. Coulibaly, Jörg Romeis, John L. Teem, Willy Tonui, Brian Lovett, Aditi Mankad, Abraham Mnzava, Silke Fuchs, Talya D. Hackett, Wayne G. Landis, John M. Marshall, Fred Aboagye-Antwi

**Affiliations:** 1 Department of Life Sciences, Imperial College London, Silwood Park, Sunninghill, Ascot, UK; 2 Centre for Environmental Policy, Imperial College London, Silwood Park, Sunninghill, Ascot, UK; 3 Independent Risk Assessor, Baarn, The Netherlands; 4 University of Exeter Business School, Exeter, UK; 5 Department of Zoology, University of Oxford, Oxford, UK; 6 Program On Science, Technology & Society, John F. Kennedy School of Government, Harvard University, Cambridge, MA USA; 7 Cambea Consulting Ltd, Reading, UK; 8 Malaria Research and Training Center (MRTC), University of Sciences, Techniques and Technologies of Bamako, Bamako, Mali; 9 Research Division Agroecology and Environment, Agroscope, Zürich, Switzerland; 10 Genetic Biocontrols LLC, Tallahassee, FL USA; 11 Environmental Health and Safety (EHS Consultancy) Ltd, Nairobi, Kenya; 12 Division of Plant and Soil Sciences, West Virginia University, Morgantown, USA; 13 CSIRO Synthetic Biology Future Science Platform, CSIRO Land & Water, Brisbane, Australia; 14 African Leaders Malaria Alliance, Dar es Salaam, Tanzania; 15 Institute of Environmental Toxicology and Chemistry, College of the Environment, Western Washington University, Bellingham, WA USA; 16 Divisions of Biostatistics and Epidemiology, School of Public Health, University of California, Berkeley, USA; 17 Department of Animal Biology and Conservation Sciences, University of Ghana, Legon, Accra, Ghana

**Keywords:** Gene drive, Population suppression, Vector control, Malaria, Environmental risk assessment, Engagement, Modelling, Ecological risk

## Abstract

Building on an exercise that identified potential harms from simulated investigational releases of a population suppression gene drive for malaria vector control, a series of online workshops identified nine recommendations to advance future environmental risk assessment of gene drive applications.

## Background

In 2020, there were 228 million cases of malaria, resulting in 602,000 deaths, reported in the World Health Organization (WHO) African region [1], underscoring the need for the development of novel interventions that can complement existing malaria control strategies [2, 3]. One such innovative approach to control malaria vectors that is currently under active investigation is the use of engineered gene drives to bias the inheritance of introduced traits and disrupt the malaria transmission cycle [4, 5]. This would involve the release of genetically modified mosquitoes (GMM) of malaria vector species, such as *Anopheles gambiae*, resulting in the introduction and propagation of a transgene via gene drive into wild vector populations. Such transgenes could disrupt malaria transmission in those populations either by reducing their densities, in the case of population suppression gene drive, or by reducing their vector competence, in population replacement gene drive [6].

Gene drive applications, both as envisaged or in development, are considered to share many of the same biosafety considerations as other genetically modified organisms (GMOs) via the characteristics of transgenesis [7, 8] and are thus subject to regulatory oversight and environmental risk assessment (ERA) under biosafety legal frameworks globally (see Box 1). However, unlike the case in other GMOs, gene drive transgenes for vector control are more likely to be designed to disperse beyond immediate release locations and persist for many years in target populations; this makes assessment of ecological risks with a broader scope of spatial and temporal considerations for gene drive applications particularly important [8, 9].

**Box 1:** ERA of GMOs

ERA involves a technical assessment of biosafety: the safe handling, transport and use of living modified organisms (LMOs, alternatively known as Genetically Modified Organisms (GMOs)) resulting from biotechnology that may have adverse effects on biological diversity, also taking into account risks to human health. ERA is a process to identify significant risks to the environment and health, estimating their magnitude and likelihood and defining any risk management that may be required. The fundamental features of ERA are consistent across different global jurisdictions [7, 8, 11–14] and consist of four key stages:
I.*Problem formulation* allows the identification of potential adverse effects (hazards) associated with the GMO. It is a systematic way of structuring the ERA at this first stage by establishing the policy context and scope via the identification of protection goals, aspects of the environment and health that are considered to be of value in the jurisdiction where the intervention is being considered and so are identified from a combination of policy, legislative, regulatory, and community priorities. These protection goals are then used to consider a broad array of potential harms from the intervention in a highly iterative, systematic approach involving a diverse range of expert input. Based on scientific evidence and data specific to the intervention, the plausibility of each potential harm to protection goals is investigated via the development of a pathway to potential harm in which the causal chain of events that would be required for that potential harm to occur is defined. Next, risk hypotheses are developed to interrogate key individual steps in that pathway. Then, an analysis plan is constructed describing the measurement endpoints to test each of the risk hypotheses, as well as other potential sources of data that might reduce aspects of uncertainty in a given pathway
II.*Exposure Characterization* identifies the likelihood that potential harms occur from identified routes of exposure
III.*Hazard Characterization* establishes the magnitude and type of harm that might be caused if it were to occur
IV.*Risk Characterization* allows determination of the overall level of risk, taking into account both exposure and hazard characterization, in order to facilitate decision-making about risk mitigation, risk management and risk communication

Therefore, before such gene drives could be considered for field release, potential impacts from the intervention, including legal issues, (e.g. potential consequences of transboundary movement), socioeconomic effects (e.g. potential changes in insecticide use) and biosafety (e.g. risks to human health and the environment), must first be identified, assessed and, where appropriate, managed [10]. Some of these issues are beyond the scope of biosafety considerations in ERA, but nonetheless can be addressed separately in other forms of assessments that are discussed further below and highlighted in Table 1.

**Table 1:** Comparison of risk and impact assessments relevant to gene drive applications

	Strategic Environmental Assessment [SEA] [16, 17]	Environmental & Social Impact Assessment [ESIA]^a^ [18]	Environmental Risk Assessment [ERA] [7, 12, 54, 55]
Purpose	Supports political and policy level decision-making	Supports decision-making on specific projects	Supports decision-making on specific products
Scope	Policy, plans and programmes	Specific projects	Specific projects
Legislative basis	National and EU environmental regulations	National and EU environmental regulations	International law and national biosafety legislation^b^
Guidance	Extensive documentation, including from WHO [75], UNEP [76, 77], World Bank [78], OECD [79, 80] and EU [81]	Extensive documentation, including from WHO [82], World Bank [78], International Finance Corporation of the World Bank Group [83], African Development Bank Group [84], UNEP [76, 77] and EU [85]	Extensive documentation, including from CBD [11], EFSA [7, 12], NASEM [9] and WHO [8]
Number of exercises envisaged	Single for a general class of intervention (e.g. low threshold gene drive for malaria control)	Single for a specific release at specific location but likely to be multiple based on specific context and geography	Likely to be multiple, arising from different sponsors and assigned assessors
Sponsor	Developer	Developer	Developer; Organization independent of developer; Regulator^c^
Assessor	Organization independent of developer	Organization independent of developer	Developer; Organisation independent of developer; Regulator^c^
Extent of engagement	Public facing and participative; Stakeholders engaged throughout process^d^; Data collection by interviews, focus groups, and desk-based work	Public facing and participative; Stakeholders engaged throughout process^d^, Data collection by interviews, focus groups, and desk-based work	Principally agency-applicant interaction; Engagement with broad experts on technical issues and parameters at discrete stages of assessment; Stakeholder engagement in problem formulation and model and scenario development^e^
Parameters	Sustainability in relation to social, economic, health, environment endpoints and cumulative impacts, examination of alternatives to proposed intervention	Environmental, social, economic, and health endpoints, drawing on SEA and ERA endpoints	Biosafety endpoints on environment and health
Analysis type	Qualitative	Qualitative	Probabilistic; Qualitative; Combination of both of the aforementioned^f^
Role of post-release monitoring	None	Monitoring and audit as set out in Environmental and Social Management Plan to mitigate negative or enhance positive impacts of application	Post-Market^g^ Environmental Monitoring to assess risks and uncertainties identified and confirm hypotheses in the ERA, including on temporal or spatial scales and reversibility [12]

^a^Also known as Environmental, Socioecomonic and Health Impact Assessment (ESHIA) or Environmental Impact Assessment (EIA) in some jurisdictions

^b^The Cartagena Protocol on Biosafety to the Convention on Biological Diversity (CBD) is an international agreement that has entered into force in 173 countries, with notable exceptions such as Argentina, Australia, Canada, Israel and the United States of America

^c^For early gene drive applications, it is anticipated that all three types of potential sponsor and assessor are likely to be involved

^d^For gene drive applications, these assessments are anticipated to take place over circa two to three years

^e^In the EU, there is a mandatory engagement with the public for all GMOs that are environmentally released. The engagement is in the form of commenting on draft decisions. In this article, in ERA of gene drive applications we recommend wider engagement of experts and publics than has previously been the case with ERAs for conventional LMOs or GMOs (see Recommendations One, Four, Seven, Eight and Nine)

^f^In this article, we recommend using a combination of probabilistic and qualitative approaches to ERA for gene drive applications (see Recommendation Seven)

^g^In the case of gene drive applications, there would likely be a monitoring requirement even if the release was for pre-market trials

As is the case for all GMO applications, regulatory oversight either derived from international agreements such as the Cartagena Protocol on Biosafety to the Convention on Biological Diversity (CBD) [15], or exclusively from national law for non-parties to those agreements, is underpinned by ERA (see Box 1). Additionally, under certain national legal frameworks, notably in the African continent, the use of GMOs is also subject to oversight under environmental laws, which allow for the potential additional conduct of Strategic Environmental Assessment (SEA) and Environmental and Social Impact Assessment (ESIA) for gene drive applications. SEA facilitates consideration of impacts from a general class of intervention and is designed to support policy and political decision-making [16, 17]. For example, the legal impacts of the potential transboundary movement of gene drive applications or impacts on the health sector broadly from a malaria intervention would probably be best suited to assessment at this level.

By contrast, ESIA is a more specific form of assessment which is suited to the implementation of specific projects such as field studies or evaluations, but also extending to interventions, such as area-wide vector control [18]. ESIA examines a much broader scope of impacts, both positive and negative, than ERA and across the areas of environment, socioeconomics, and health. As gene drive applications have the potential for far-reaching impacts across disciplines, the use of ESIA tools and methodologies, such as around stakeholder engagement and the management of environmental and socioeconomic impacts, are considered to be particularly appropriate for gene drive applications.

As with ESIA, ERA is *project*-specific. For example, it involves a characterization of the release location and area of transgene dispersal. However, unlike ESIA, ERA is also *product*-specific as it encompasses a technical assessment of biosafety risks to the environment and health from the intervention itself (see Box 1) [8, 10, 15, 19]. In the biosafety context, ERA can thus be thought of as a module in the wider framework of impact assessments, feeding in information about risk to defined protection goals. ERAs will, therefore, play an important part in the appraisals of the potential use of gene drive applications in the field, but only if integrated alongside other evaluations of predicted efficacy, reliability, costs, logistics, socioeconomic and stakeholder considerations [8–10].

Regulations on the ERA of GMO applications, including GMMs, particularly those incorporating the Cartagena Protocol on Biosafety into national legislation [15], aim to ensure an appropriate quality of evidence to support decisions through risk assessments, allowing both quantitative and qualitative methods depending on the circumstances [7–9, 11, 12]. Commonly, the design of an ERA is articulated in the form of guidelines, which establish the steps to follow when conducting an ERA and provide guidance both to gene drive developers preparing ERA and to risk assessors evaluating them [8, 11, 20].

Unlike SEA, and depending on the GMO involved, there could potentially be multiple ERAs for the same jurisdiction that would be conducted independently of one another and by different risk assessors for GMO applications, including for gene drive applications (see Table 1). This should allow a plurality of analytical approaches and viewpoints to be considered.

For the simulated investigational releases of a non-localized, self-sustaining population suppression gene drive for malaria vector control in West Africa, a problem formulation approach was recently followed to identify potential harms associated with the release and the plausible pathways by which such harms could occur [21] (see case-specific example in Box 2). This allowed the construction of analysis plans to identify evidence and generate data and that could inform the subsequent stages of this ERA, including hazard and exposure characterization [12]. Following publication of the problem formulation, a series of workshops was held to advance ERA for gene drive applications, using as a specific use case a mosquito strain being developed for population suppression gene drive for malaria control in West Africa [21] (see Box 3). As mentioned above, some potential concerns around the use of gene drive are beyond the biosafety focus of ERA and are likely to be addressed in other types of assessment.

**Box 2:** Problem formulation of the specific use of a population suppression gene drive for malaria vector control in West Africa

A CRISPR-Cas9 transgene homing at the *doublesex* locus (*dsxF*^*CRISPRh*^) causes homozygous transgenic females to be both sterile and non-biting [31]. When introduced into both small and large cages of laboratory populations of the malaria mosquito vector *Anopheles gambiae*, the transgene increases rapidly in frequency, ultimately causing those populations to collapse [31, 74]. Drawing on protection goals identified from a series of consultative workshops in Africa [38], problem formulation as a first stage in an ERA (see Box 1) was used for simulated investigational releases of the *dsxF*^*CRISPRh*^ transgene carrying mosquito in West Africa [21, 48], where the transgene would be expected to act as a non-localized, self-sustaining gene drive. Plausible pathways to potential harm were developed that described the cause-effect chain of events that could lead to 46 discrete potential harms, such as increased disease transmission in humans or reduced ecosystem services [21]. For each pathway, risk hypotheses were developed that would allow future investigation of key individual steps, drawing on data and evidence gathered from an analysis plan to test each of those individual risk hypotheses, in subsequent stages of ERA

**Box 3:** Format of workshops on ERA for gene drive applications

To inform and advance ERA for gene drive applications, 50 experts from Africa, Oceania, Europe and North America representing an array of disciplines including risk assessment, biosafety regulation, modelling, population genetics, social science, ecology, entomology, vector control and molecular biology participated in a series of six online workshops held between 27th April and 7th May 2021. The opening workshop presented key findings from the problem formulation on the specific use of a population suppression gene drive for malaria vector control and framed the overall objectives of the workshops. The next four considered approaches to optimize future ERA for gene drive applications, focussing in particular on engagement, technical aspects of conducting ERA and evaluation of potential ecological risks. The final workshop synthesized the discussions and outputs from the previous five. A subset of participants, the authors here, volunteered to translate the outputs from those workshops into a tangible record of those proceedings with specific recommendations as outlined in this article	

Therefore, the workshops also considered ERA within the wider context of SEA and ESIA, where engagement was a cross-cutting theme. The outcomes of those workshops led to the development of nine recommendations for ERA for gene drive applications for malaria vector control. One is on guidance around engagement in ERA for gene drive applications, another on inclusive engagement in problem formulation, while a third is on engagement in the wider context of governance and policy decision-making for vector control. The six remaining recommendations are focussed on technical aspects of ERA for gene drive applications, although engagement considerations were also inherent to two of these.

### Recommendations to advance ERA for gene drive applications

Engagement represents a cornerstone of effective ERA for gene drive applications for malaria vector control [8]. Who to engage and how are of critical importance. The identification of some stakeholders as “experts” could create perceptions that their inputs are more informed or valuable than those of others who might nonetheless provide critical contributions based on their own lived experiences. By contrast, an equitable differentiation can be made between stakeholders who are “technical experts”, with deep knowledge and proficiencies directly applicable to the operational conduct of ERA, and “non-technical experts” with broader levels of experience that can contribute to the overall specifications of the ERA.

The development of commonly-accepted guidance for ERAs of gene drive GMOs has been and remains critical as it will inform how an ERA is conducted for any given gene drive application. Engaging a range of actors with broad expertise in guidance development is recognized by many as crucial in establishing the credibility of guidance and its general use. Emphasizing this point, provisions for engagement in ERA of GMOs are included in the Cartagena Protocol on Biosafety [11, 15] and the 2019 draft report on risk assessment for gene drive CBD Secretariat calls for public consultation [22]. Echoing this call for engagement, the WHO has also recognized the importance of engagement and how it might strengthen the ethical foundation of research, technical and public health goals in its recent guidance framework for testing genetically modified mosquitoes [8]. It therefore follows that engagement should be an essential element in the development of guidance on ERA of gene drive applications.

Meaningfully moving from broad statements calling for engagement in ERAs with actors who hold wider expertise to specific ERA designs is challenging. For example, the 2016 report from the National Academies of Sciences, Engineering and Medicine (NASEM) on gene drives [9] has two separate chapters on engagement and risk assessment. While these chapters cross-reference each other to some extent, there is little guidance on how engagement might be integrated into ERA guidelines for gene drive organisms.

Incorporating specific recommendations within ERA guidance documents on how to structure engagement could help connect these broad goals to specific ERAs. Regulators are currently considering the adequacy of regulations and ERA guidance for gene drives in advance of potential field trials. For example, in 2020, the GMO Panel of the European Food Safety Authority (EFSA) advised the European Commission that its existing guidance for the risk assessment of GM animals was adequate, but that further specific guidance was needed for gene drive applications in insects for vector control [7].

Guidance could, for instance, specify who to involve, when to involve them, as well as why and how uncertainty should be addressed [23–25]. More specific guidance on engagement in ERA might also support the translation of international instruments into national legislation and regulations in order to accommodate local and regional differences in circumstances, values, literacy levels and technology access [22, 26]. For example, while Article 23 of the Cartagena Protocol on “Public awareness and participation” obliges national governments to engage with ‘the public’ on GMO applications [15], there appears to be a gap in guidance on how this can be achieved effectively [11].

Although national guidance often includes a public comment period on a draft document, it can be insufficient as a form of engagement [9]. Broadening the range of experts in engagement would benefit the development of guidance for ERA of gene drive applications where there are more uncertainties than in more established GMO approaches. Three ways that expertise could be meaningfully broadened in the development of guidance include:

i. Involvement of a plurality of perspectives and expertise *within* individual disciplines to help risk assessors identify and interrogate gaps and assumptions in the ERA that had not been previously considered;
ii. Inclusion of relevant expertise from *across* different disciplines, including the social sciences, natural sciences, humanities, and engineering, should be considered [27], as expertise from disciplines that are not traditionally included in the development of guidance or individual ERAs could help to bring a broader range of perspectives and expertise to the process and encourage the identification and interrogation of gaps and assumptions;
iii. Consideration of the perspectives and expertise of members of stakeholder groups beyond the above disciplines; expanding the pool of experts for guidance development of ERA for gene drive applications could increase the legitimacy of the overall assessment process [28]; these may include potentially impacted parties and wider publics to help to inform protection goals and decision-making criteria for problem formulation [7].

Development of guidance of ERA for gene drive under the auspices of the WHO, CBD, African Union, or an amalgam thereof, would enjoy the confidence of a wide body of stakeholders across different jurisdictions. For example, the recent West African Integrated Vector Management (WA-IVM) initiative, involving the African Union Development Agency (AUDA-NEPAD) and West Africa Health Organization (WAHO) could provide a suitable platform.

**Recommendation One: Additional guidance for ERA of gene drive applications should be developed by a broad range of actors.**

Clear articulation of the intended outcomes of an intervention is essential to evaluating any potential unintended consequences and harms. Defining the Target Organism (TO) of an intervention is therefore a fundamental element in the ERA of a GMO. In Connolly et al. [21], problem formulation (see Box 1) was conducted for a population suppression gene drive proposed for the control of the human malaria vector *Anopheles gambiae *sensu lato (s.l.), a complex of nine species, six of which are known to vector human malaria (see Box 2). Hybridization between sibling species of the complex can yield fertile hybrids [29, 30], which coupled with conservation of the *doublesex* target sites for the CRISPR-Cas9 nuclease amongst sibling species [31], led Connolly et al. [21] to define the TO as all nine species of the complex. However, including non-vector species of a complex as part of the defined TO set exceeds the intention to specifically target vectors. Therefore, the definition of TO in ERA of gene drive in species complexes requires more nuanced consideration and further refinement than would be the case for more conventional GMO applications [7, 32].

Indeed, EFSA [7] has already acknowledged that for gene drive applications the TO might include an individual population, a single species, or a species complex defined as a set of partially reproductively connected species. Therefore, the TO should be defined by the applicant in relation to the intended outcomes of the gene drive application. Thus, depending on how the TO is defined, intended outcomes might differ across the spectrum of a species complex [7].

Further considerations for gene drive applications might include clear (i) articulation of the intended impact on the TO populations in the wild, (ii) definition of what negative impacts the introduction of the transgene into the TO has in order to make it a justifiable target and (iii) description of how a trait affects different members of a complex in ways that would influence its efficacy.

**Recommendation Two: The definition of the term ‘Target Organism’ for gene drive applications involving species complexes requires more nuanced consideration than for other GMO applications**.

This problem formulation approach is widely used in ERA of GMOs and sets the foundation for the overall risk assessment process by framing the issues that are specific to the case under assessment [11, 33]. A crucial step in problem formulation is to define what environmental and health resources need to be protected under the relevant national policy regulations, or ‘protection goals’ (see Box 1 ), and what qualifies as harm to these resources [34, 35]. Stakeholder input into what is considered to be of value in the environment and to health is also essential. In the next step of problem formulation, plausible pathways to harm are constructed to describe how the proposed activity could lead to possible harm to those protection goals. A pathway to harm is in effect a causal chain of events required for a harm to be realized [36, 37]. Each step in the pathway provides an opportunity to formulate risk hypotheses that can then be tested to characterize that specific risk [33]. The problem formulation approach thus provides both flexibility and a fit-for-purpose framework for case-specific risk assessments.

In Connolly et al. [21], problem formulation addressed the simulated investigational releases of a specific *doublesex*-based population suppression gene drive to control the human malaria vector *An. gambiae* in West Africa and considered plausible pathways to harm based on broad protection goals that had been defined in a series of consultations with policymakers and regulators in Africa [38]. Although the problem formulation approach is considered fit-for-purpose for the assessment of this specific population suppression gene drive application [7], high-level, broad protection goals can sometimes be in conflict with each other when not distilled into more precise and specific *operational* protections goals [35]. For example, efficient suppression of malaria vector species could be considered a potential harm to biodiversity (loss of insect species) to some stakeholders, but a potential benefit to human health (reduced malaria transmission) may be more consistent with public health policy outcomes and prioritized by other stakeholders.

It has previously been recognized that protection goals outlined in legislation are often too broad to be directly applicable for environmental risk assessment [33, 35, 39, 40]. Establishing operational protection goals for individual pathways to harm thus represents an important area for future development of ERA for gene drive approaches to malaria vector control [32].

**Recommendation Three: ERA for gene drive applications should be founded in a problem formulation approach and addressed using specific operational protection goals**.

One of the reasons problem formulation is a key step in structuring the ERA is because it draws together values that underpin policy aims, protection goals, assessment endpoints, and methodology to define the problem and approach for the overall risk analysis [25, 41] (see Box 1). While engagement is also needed in model and scenario development (see Recommendations Seven and Eight), effective engagement at the problem formulation stage of ERA is essential to ensure the ERA is robust and adequate in scope.

Considerations on the potential impacts of localized self-limiting gene drives are likely to differ substantially from those of non-localized, self-sustaining interventions [6]. Therefore, the process of engagement during the ERA, including the type of actors and expertise with whom to engage, will be case-specific to reflect those different purposes in individual gene drive applications. However, engagement should involve stakeholders with diverse backgrounds and expertise but should especially be inclusive of those communities where the intervention is being considered for use, as they will contribute unique and essential perspectives and expertise to the ERA for a gene drive application [24, 26] (see Recommendation 1). Ideally, such engagement would come before initiation of an ERA so that relevant values, knowledge, and experience informs the design from the outset of the plan being assessed and the data collection for the regulatory dossier that is ultimately to be submitted to risk assessors.

**Recommendation Four: Engagement, specifically in the problem formulation stage for ERAs of gene drive applications, should include actors with broad expertise**.

The realistic worst case scenario (RWCS) is a generally used concept in risk assessment and management [42, 43], representing the most severe outcome that can realistically be envisaged to occur in a given situation based on the best available evidence at the time. The RWCS is one of the most commonly used approaches to come to a conservative risk estimate and is applied in a wide range of fields [44, 45]. In ERA of GMOs, RWCSs are typically used in qualitative risk assessment in cases of high uncertainty or where there is a lack of data available at the time of the assessment. They are frequently used in early stages of ERA with respect to exposure and the consequences of exposure to focus on substantive risks that warrant further consideration. Where a potential harm is identified at high exposure under such worst case assumptions, it can then be determined if this would remain under more likely scenarios.

Conversely, should no or minimal harm be identified under RWCSs, it can reasonably be concluded that greater harm is unlikely to occur under more realistic scenarios [41]. Such a tiered approach is also generally applied to experimental testing; potential harm is evaluated within different tiers that progress from worst-case exposure scenario conditions, in controlled laboratory environments, to more likely scenarios under semi-field or field conditions [46, 47]. The RWCS approach can also be used to focus on steps in specific pathways to harm that can be tested most rigorously. Where these steps are evaluated as unlikely to occur, particularly under worst case conditions, this would interrupt the causal chain of events for that specific pathway and may lead to its rejection as a potential source of harm [36]. In cases where the likelihood of a potential harm occurring cannot be ruled out even under more realistic scenarios, risk management measures can be proposed to address uncertainties and to mitigate potential effects*.* For example, uncertainty may exist on the actual level of suppression, or its variability, at release sites for the gene drive mosquitoes in West Africa [48]. Therefore, a starting point in testing risk hypotheses in pathways to harm could be to assume the most extreme entomological efficacy scenarios of either 100% or 0% suppression of populations of *An. gambiae*.

The RWCS could also be used in evaluating the spatial scale of the receiving environment in the ERA. Although Connolly et al. [21] described investigational releases of the population suppression gene drive that were limited to West Africa, the released transgene could theoretically disperse to any areas where the TO naturally occurs, defined in that context as the entire species complex of *An. gambiae*, and which would therefore include most of sub-Saharan Africa. In the absence of data to prove otherwise, testing of risk hypotheses could involve considering this broader receiving environment spanning the entire geographic range of *An. gambiae* as a RWCS. The same RWCS approach could also be used to consider the temporal scale of exposure to the transgene which, for example, could be postulated to persist indefinitely in a population of sexually compatible species in the receiving environment. A further example of a RWCS approach could also be to consider extreme climatic or seasonal conditions across Africa. For example, El Niño climatic fluctuation events recur every two to seven years and tend to cause drought in Southern Africa and excessive rainfall in East Africa [49], thereby potentially impacting the availability of aquatic habitats for malaria vectors including of the *An. gambiae* species complex.

Such use of RWCSs involves the application of extreme situations to the evaluation of specific pathways to harm in the problem formulation that may require relevant expertise from a range of different disciplines, prioritizing representation of those disciplines from geographic regions and contexts within the RWCSs. This could be clarified in guidance for ERA of gene drive (see Recommendation 1).

**Recommendation Five: Use of ‘realistic worst case scenarios’ should be considered when testing risk hypotheses in pathways to harm in ERA for gene drive applications**.

Any potential harm identified in the ERA of gene drive in mosquitoes should be considered for assessment in a comparative way, that is compared to both the effects from a predefined comparator such as a non-GMO *and* the context in which they used and managed [7, 8]. For example, comparisons could be considered to assess whether the gene drive application is more or less harmful than other currently deployed malaria vector control measures, such as insecticide-based applications that are used to manage populations of wild-type mosquitoes in disease control programs [8]. Such interventions thus represent one level of comparator to gene drive applications, to inform ERA and contextualize risks [7, 8, 32, 50].

On another level, comparisons could be made between the transgenic strain and its non-transgenic background strain to detect intended, or unintended, changes at molecular or phenotypic levels. The latter comparison is conceptually similar to the established assessment of GM plants, for which extensive experience exists [51–53]. The choice of comparators from the range of possibilities will depend on the specific risk hypotheses being considered. Multiple comparators will be used in the ERA for a given gene drive application, reflecting specific aspects of individual pathways to harm and risk hypotheses. This will allow a comprehensive and integrated assessment of potential risks resulting from gene drive applications.

**Recommendation Six: A range of comparators should be considered in ERA for gene drive applications in order to contextualize risks**.

ERA for gene drive applications should be conducted on a case-by-case basis. Therefore, the precise approach adopted for an ERA will depend on the nature the intervention, availability of data, levels of uncertainty and the nature of risks being assessed. Throughout the development of gene drive applications, ERA will be an iterative process in which cause-effect chains are assessed, then re-assessed with risk management plans adjusted towards lowering the likelihood that any identified harms would occur.

Probabilistic risk assessments use quantitative modelling approaches to represent a probability distribution for a range of potential outcomes for a particular event [54–60]. Qualitative risk assessments categorize, in a structured and systematic way, the likelihoods and consequences of outcomes into a limited number of ordered classes to give a categorical indication of relative risk, such as 'high', 'moderate', 'low' or 'negligible' [11, 12]. Where feasible, such qualitative terms should preferably be defined as precisely as possible in a quantitative way [11, 12]. However, some potential risks may be best communicated in qualitative terms, especially when drawn from stakeholder concerns that might also have been initially articulated in a qualitative manner, such as "Could the modified mosquitoes make me sterile?".

In reality, complex ERAs are unlikely to be either fully 'probabilistic' or 'qualitative' and could involve a combination of these approaches. Some risk assessments that might be termed qualitative, for instance, may also include quantitative analyses and mathematical modelling in the evaluation of some risk components. In risk assessments that are termed probabilistic, the use of Bayesian networks can ensure that the input and output will be categorical so that risk can be indicated both numerically and by a discrete number of categories. This range of approaches to risk assessment can be used to illustrate the considerable uncertainties involved in predicting outcomes from events where there is little available information and results in a more robust enquiry into the overall risk.

Modelling is a key tool in describing relationships in a cause-effect chain that can underpin ERA for gene drive applications [7, 9, 10, 55, 61]. Models can inform both quantitative and qualitative risk assessments, depending on the levels of uncertainty and the structure of the model. Scenarios describe planned events, and models of the subsequent processes and outcomes, harmful or otherwise depending on the social values applied, and offer predictions for the risk assessment. Both model and scenario development should include input from a broad range of actors, such as those in Recommendations One and Four, as wider expertise has been shown to improve their quality [27]. While models used in ERA might be expected to require extensive data, even conceptual models may be helpful in the continuous process of evolving the assessment of risk in the emerging designs of gene drive applications or processes. Scenarios and models are also very useful in establishing a structural framework for expert and decision-maker elicitations of relevant experience or opinions on processes that could lead to harm. Developers will need to be able to articulate plans, for example the traits in a gene drive application and the protocols for releases, which can be structured in some form of model to predict outcomes.

**Recommendation Seven: ERA for gene drive applications should draw on the range of probabilistic and qualitative analyses, depending on data availability, levels of uncertainty, and the nature of the risks being assessed**.

Ecological interaction networks map the connections linking species within ecosystems [62, 63]. In network terminology, individual species (or groupings comprising multiple similar species, perhaps defined by their functions) are referred to as ‘nodes’, and the interactions between them as ‘links’ or ‘edges’. Edges can represent a variety of interspecific interactions, including predation, resource use or mutualism. For ERA, the relevant composition, and temporal and spatial scale of such a network would vary according to the intervention, nature of the release and risk being assessed. These networks can be used in two main ways.

First, documenting the network of interactions within ecological communities provides an opportunity to track how gene drive applications might impact on other species connected directly or indirectly to the TO thereby identifying component species that might alter in abundance. For example, if the TO for a population suppression gene drive is a predator, any species directly linked to it as prey may experience reduced predation, and perhaps an increase in abundance. Ecological network analysis also allows the tracking of indirect interactions through, for instance, shared resource use, as well as defining the role of species in a community [64]. Depending on the identity of those connected species, their functional role and their ‘centrality’ within a network [62], such an outcome may or may not be considered as harmful within the framework of an ERA. Ultimately, all species within ecological communities will be inter-linked via such chains of interactions, but ecological principles suggest that those that are more intimately linked, in terms of the proximity, specificity and frequency of interactions, are those most likely to be impacted by gene drive applications [62].

Second, the structure of interaction networks and ecological communities that comprise them can be described using a variety of summary metrics, some of which have been shown to be linked to desirable dynamic properties of those communities, such as stability and resilience [63, 65]. While acknowledging that non-equilibrium processes and spatial heterogeneity are often important in structuring ecological systems [66, 67], ERAs can evaluate whether gene drive targeted to component species within networks will yield structural shifts in networks that are likely to lead to state changes in the structure and functioning of the community that would have negative value for stakeholders.

Complex ecological network models in principle allow probabilistic predictions of the direct and indirect effects of interventions such as gene drive, but this requires detailed and ideally quantitative data on the pre-intervention network structure [68, 69]. One approach to characterize the structure of complex interaction networks is a combination of intensive community-wide genetic barcoding and metabarcoding. This is being used in the context of *An. gambiae*, where the DNA of invertebrate species that co-occur with these mosquitoes as larvae or as adults is sequenced, as well as the gut contents or faeces of species that might consume them. To date, data of this type that could directly inform ERAs have rarely been available and may be specific to a limited spatial and environmental context. A further challenge is that networks are highly dynamic through time [62], with the abundances of component species tending to vary markedly, particularly for short-lived organisms with ‘fast’ population dynamics such as many insect species [70]. Stochastic variations in abundances of component species within networks will be superimposed on often pronounced, albeit relatively predictable, seasonal variations. However, there is evidence that the majority of community functioning is maintained by a "core" of that community so that, even with limited sampling, the functional core of the community can be identified. For instance, Hegland et al. [71] showed that 20% of sampling captured 70–85% of the most functionally important species, supporting the notion that dynamic processes are potentially less important in the maintenance of functional communities [64].

Community-wide consequences of a gene drive intervention could also be assessed based on groupings of species and a broader understanding of the likely flows of energy and impact between nodes defined not at the species level but in terms of functionally and trophically similar taxa within networks. This could usefully be combined with an experimental approach to test RWCS of impacts of interventions, for example in cages or mesocosms. In these cases, network data can be valuable to identify the species most likely to be affected directly by the intervention, as well as those that might 'rewire' their interactions within networks as the availability of resources changes [63]. Data on the natural or pre-intervention range of variation of species’ abundances and the structural properties of the networks that they are embedded within can help to put potential ecological risks in context. Risk assessors will need to ask whether the RWCS outcomes predicted following a gene drive intervention fall within the range of states that are observed under pre-intervention conditions.

While technical experts, from amongst developers and via independent risk assessment groups, are required to construct and validate appropriate models of the processes that could lead to harm, a wide range of stakeholders who are nontechnical experts, such as those described in Recommendations One and Four, should also participate in determining acceptable combinations of scenario plans and model representations of potential harm [27] (see also Recommedation Seven). This entire process will be central to engagement on the ERA, ensuring there is widespread and deep understanding of, and input into, what is being proposed, how this could lead to ecological harm, and how those harms can be quantified or classified in ways that sufficiently inform technical, regulatory and societal decisions.

**Recommendation Eight: ERA of potential ecological risks from gene drive applications should use concepts of ecological interaction networks to assess the impacts on dynamic properties that have been defined as important on the basis of biological considerations and stakeholder values**.

As outlined in the Background section, ERA is one component in the wider framework of impact evaluations, providing information on potential risks to defined protection goals in the context of biosafety. ERA thus plays an important part in assessment of the potential use of gene drive applications in the field, but only if integrated alongside other evaluations of predicted efficacy, reliability, costs, logistics, socioeconomic and stakeholder considerations [8, 9, 15]. Within these evaluations, engagement should not be considered as a solitary event, but rather as an iterative process that engenders multi-level dialogue and information sharing.

Complementary engagement in guidance development, problem formulation in ERA and related impact assessments such as ESIA should create a better understanding of assumptions being made and incorporate knowledge about how a technology might respond to and ‘fit’ within, and interact with, its intended social and ecological environment [27]. Key stakeholders can help to contextualize these types of place-based issues as they relate to the intervention at hand. Like engagement for ERA, engagement as part of another type of assessment should not be considered as a single event, but rather a multitude of inputs that should go into the design of plans, protocols, and studies well before the actual ERA or ESIA is carried out. Engagement early in this process is likely to have the greatest impact on how appropriate, legitimate and acceptable the designed product and its use ultimately will be.

Engagement will also be important in broader risk governance decision-making at sovereign levels, such as via national regulatory agencies. Risk governance decisions contribute to legitimate political risk decision-making and are concerned with levels of acceptable risk, as well as managing and communicating risk [26, 72]. Broader societal engagement will also be needed in national policy-making contexts where decisions about the desirability of particular gene drive applications can be debated in the context of alternative approaches to malaria control. High levels of inclusivity require relevant actors to participate and contribute effectively and for their contributions to be implemented in regulatory decisions that represent both technical and social problem structuring, inputs, and decision processes.

In order to achieve relevant and effective implementation of engagement for gene drive applications, there may be a need for innovative engagement mechanisms that help to achieve efficient understanding of relevant values, knowledge and experience from all those who may be able to contribute to the assessment processes (See Table 1). Additional, inclusive fora for engagement on gene drive applications involving a comprehensive range of stakeholders and publics will be needed within these broader decision-making contexts. These additional engagements will be needed to help decision-makers and societies determine whether or not particular gene drive products should be released. Such engagements should also maintain alignment of technology and its development within wider societal needs [73].

**Recommendation Nine: Engagement in ERA for gene drive applications should complement engagement in (i) related impact assessments, (ii) risk governance frameworks and (iii) national policy-making contexts**.

## Conclusions

Following publication of a problem formulation for investigational releases of a population suppression gene drive for malaria vector control (See Box 2) [21], participants in a series of online workshops developed nine recommendations to advance future ERA for gene drive applications in general and the case-specific application in particular (see Table 2).

**Table 2:** Recommendations on ERA for gene drive applications

1.Additional guidance for ERA of gene drive applications should be developed by a broad range of actors
2.The definition of the term ‘Target Organism’ for gene drive applications involving species complexes requires more nuanced consideration than for other GMO applications
3.ERA for gene drive applications should be founded in a problem formulation approach and addressed using specific operational protection goals
4.Engagement, specifically in the problem formulation stage for ERAs of gene drive applications, should include actors with broad expertise
5.Use of ‘realistic worst case scenarios’ should be considered when testing risk hypotheses in pathways to harm in ERA for gene drive applications
6.A range of comparators should be considered in ERA for gene drive applications in order to contextualize risks
7.ERA for gene drive applications should draw on the range of probabilistic and qualitative analyses, depending on data availability, levels of uncertainty, and the nature of the risks being assessed
8.ERA of potential ecological risks from gene drive applications should use concepts of ecological interaction networks to assess the impacts on dynamic properties that have been defined as important on the basis of biological considerations and stakeholder values
9.Engagement in ERA for gene drive applications should complement engagement in (i) related impact assessments, (ii) risk governance frameworks and (iii) national policy-making contexts

Engagement was a cross-cutting theme running through all of these workshops. The importance of engagement is reflected in it being an integral aspect of five of the recommendations here. Recommendation One emphasizes the importance of inclusion of a broad range of expertise to develop guidance that specifies who to engage, when to engage them and why, while Recommendation Nine is centred on embedding inclusive and effective engagement in the broader framework of gene drive governance. Recommendation Four highlights the need for a broad range of expertise in engagement during problem formulation. Recommendations Seven and Eight are based on the need for stakeholder input on the development of scenario plans and model representations in ERA for gene drive applications.

Six recommendations relate to the technical conduct of ERA for gene drive applications. Recommendation Two addresses how the definition of the term ‘Target Organism’ in the context of a complex of species, such as *An. gambiae*, needs more nuanced consideration than for other GMO applications. Recommendation Three highlights the importance of using problem formulation that is based on specific operational protection goals. Recommendation Five proposes the use of the concept of ‘realistic worst case scenario’ to interrogate risk hypotheses in plausible pathways to potential harm. Recommendation Six speaks to the importance of using a range of comparators, from the levels of genotype to intervention type, in ERA for gene drive applications so that risk can be contextualized. Recommendation Seven indicates the desirability of adopting both probabilistic and qualitative approaches to ERA for gene drive applications, while Recommendation Eight stresses the importance of developing interaction networks for assessment of ecological risks that have been informed by both biological considerations and stakeholder values. In both of these recommendations, it is recognized that engagement is needed in model and scenario development in modelling evaluations.

While Recommendation One is aimed at those responsible for producing guidance on ERA, such as decision-makers like national regulators or policymakers in governments or international agencies, the eight other recommendations here are relevant to developers, decision-makers and policymakers alike. It is hoped that these recommendations can therefore contribute to the overall development and successful evaluation of innovative solutions in general, and gene drive in particular, for malaria vector control. Moreover, the set of recommendations articulated here should help to inform the ERA for gene drive applications more generally.

## Data Availability

Not applicable. All data and materials are presented in the manuscript.

## Abbreviations

AUDA-NEPAD: African Union Development Agency
CBD: Convention on Biological Diversity
EFSA: European Food Safety Authority
ERA: Environmental risk assessment
ESIA: Environmental and socioeconomic impact assessment, also known as Environmental, socioeconomic and health impact assessment (ESHIA) or Environmental Impact Assessment (EIA) in some jurisdictions.
EU: European Union
FNIH: Foundation for the National Institutes of Health
GM: Genetically modified
GMM: Genetically modified mosquito
GMO: Genetically modified organism
ISBR: International Society for Biosafety Research
LMO: Living Modified Organism
NASEM: National Academies of Sciences, Engineering and Medicine
OECD: Organisation for Economic Co-operation and Development
RWCS: Realistic worst case scenario
SEA: Strategic environmental assessment
TO: Target Organism
UNEP: United Nations Environment Programme
WAHO: West Africa Health Organization
WA-IVM: West African Integrated Vector Management initiative
WHO: World Health Organization
